# A Gel Formulation Containing a New Recombinant Form of Manganese Superoxide Dismutase: A Clinical Experience Based on Compassionate Use-Safety of a Case Report

**DOI:** 10.1155/2016/7240209

**Published:** 2016-08-17

**Authors:** Lucia Grumetto, Antonio Del Prete, Giovanni Ortosecco, Antonella Borrelli, Salvatore Del Prete, Aldo Mancini

**Affiliations:** ^1^Department of Pharmacy, University of Naples Federico II, 80131 Naples, Italy; ^2^Department of Neurosciences and Reproductive and Dentistry Sciences, University of Naples Federico II, 80131 Naples, Italy; ^3^Molecular Biology and Viral Oncology Unit, Department of Experimental Oncology, National Institute of Cancer, IRCCS Foundation, Naples, Italy; ^4^Leadhexa Inc., QB3-UCSF, San Francisco, CA, USA

## Abstract

*Background*. We report a case of bilateral posterior subcapsular cataracts (PSCs) in a 24-year-old man with an allergic conjunctivitis history caused by a long-term therapy with glucocorticoids.* Case Presentation*. The patient showed a visual acuity of 9/10 for both eyes. He followed a therapy with ketotifen and bilastine for four years. During the last six months before our evaluation, he was treated with chloramphenicol and betamethasone, interrupted for onset of cataracts and increased intraocular pressure. We treated him with ophthalmic gel preparation containing a new recombinant form of manganese superoxide dismutase (rMnSOD) at a concentration of 12.5 *μ*g/mL, only for the right eye, while left eye was treated with standard protocol of Bendazac-lysine g 0.5.* Conclusion*. This case report shows the protective effects of rMnSOD versus PSC disease, probably due to the capacity of rMnSOD of countering free radical species.

## 1. Background

The occurrence of cataracts is one of the leading causes of visual impairment in the elderly [1]. The disease can be classified as cortical, nuclear, and posterior subcapsular according to the location of the opacity within the lens [2]. The cataract is a complex disease with an etiology not completely understood [3] and related to some environmental components, including UV light, sun exposure, vitamin C deficiency [4], and some drugs. Indeed, glucocorticoids may cause steroid-induced posterior subcapsular cataracts (PSCs) being capable of inducing changes into the transcription of genes in lens epithelial cells [5]. Oxidative stress has long been recognized as an important mediator of pathophysiology in lens epithelial cells (LECs) and also plays an essential role in the pathogenesis of cataract [6]. There are a plethora of works aimed at demonstrating the importance of maintaining a proper intake of antioxidants and that indicate how genetic polymorphisms associated with genes for glutathione, a molecule reducing endogenous, may influence the development of cataracts [7]. Furthermore, some studies have been oriented to the investigation of biomarkers of oxidative stress of the cells of the lens such as higher levels of telomerase activity, and several substances were tested acting as scavengers against Reacting Oxygen Species (ROS) and lipid peroxidation, both mechanisms of cellular deterioration [8].

Recent studies have reported the association between ROS induced DNA damage of LECs and the development of cataract, indicating that oxygen free radical generators such as hydrogen peroxide can accelerate the biomolecular mechanisms that underlie the development of congenital cataract due to specific mutations [9]. The effects of topical administration of glucocorticoids on rabbit lenses are well described in animals [10]. The mechanism underlying steroid-induced damage could be due to a conformational change of lens crystallins which results in an unmasking of –SH groups with a consequent increased susceptibility to oxidation; the hypothesis could be that the protective effect exerted by some substances occurs by counteracting this oxidation.

In our previous work [11], we demonstrated the protective effect of rMnSOD against UV rays, powerful free radical generators, on the epithelium both of conjunctiva and cornea of rabbit eyes. In this case report, we reported a clinical experience based on compassionate use-safety report of the gel formulation containing the rMnSOD protein on a young patient with PSCs, caused by a long-term therapy with glucocorticoids, monitoring his clinical condition over time.

## 2. Case Presentation

### 2.1. Patient and Operative Details

An Italian 24-year-old man with an allergic conjunctivitis history was admitted to our department with PSCs diagnosis of both eyes through slit-lamp examination, caused by a glucocorticoid therapy lasting six months, one drop twice daily for both eyes. At the moment of our observation, the patient had visual acuity of 9/10 valued with a decimal system eye test card (Sbisà, Firenze) for both eyes. The patient has been treated with ketotifen 0.05% eye drops, one drop in both eyes three times a day, and ketotifen 0.05% ophthalmic gel, one drop in both eyes at evening, and bilastine 20 mg tablets per os one time in a day at the evening. He had undergone this therapy for 4 years. Furthermore, only for the last six months, he took eye drops consisting of chloramphenicol 0.5% and betamethasone 0.2%, one drop in both eyes two times a day. The patient showed an enhanced intraocular pressure (IOP), about 23-24 mmHg by a Nidek NT 2000 auto noncontact Tonometer.

**Figure 1:**
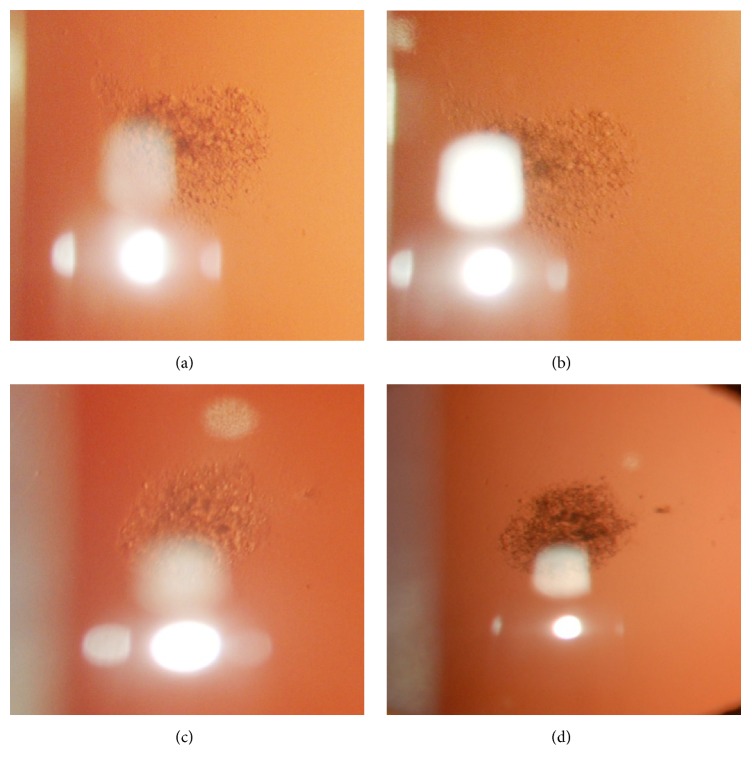
Lenz of right eye (a) and left eye (b) before the administration, respectively, of rMnSOD gel formulation in the right eye (c) and left eye (d) after the treatment with Bendazac-lysine g 0.5 eye drops.

In order to discontinue glucocorticoid therapy, the patient was treated with local specific immunotherapy; he was allergic to grass and olive tree; therefore, he was treated with a sublingual vaccine for systemic therapy and for topical therapy with eye drops containing allergens of grass and olive oil as reported in literature [12]; thus, in this way he was able to suspend glucocorticoid therapy. Figures 1(a) and 1(b) show the right and left eyes of the patient valued with the lift lamp at the moment of his admittance. In accordance with the Declaration of Helsinki (1964), it was possible to administer him for compassionate use. The ophthalmic gel preparation contained rMnSOD at a concentration of 12.5 *μ*g/mL, one drop three times a day for the right eye, while the left eye was treated with Bendazac-lysine g 0.5 eye drops, one drop three times in a day, for a period of five months. The experiment was conducted with the human subject's understanding and consent. The ophthalmic gel preparation with rMnSOD was obtained as described in the literature [11] at a concentration of 12.5 *μ*g/mL prepared as a single dosage form and in a sterile room under laminar flow hood. The developed formulations were primarily evaluated for clarity by visual observation against a black and white background in a well-lit cabinet, drug content by UV spectrophotometry at 280 nm (Shimadzu UV-visible spectrophotometer, Japan), and pH (Crison Instruments digital pH meter, Spain).

## 3. Results and Discussion

The follow-up was carried out with Digital Photo Set Righton RS-1000, an inherited full-fledged zoom photo slit lamp with 6 megapixel high resolution digital images, to monitor the slightest symptoms to the use of the programmed flash illumination. Overall zoom ratio covering 7.5x to 32.3x, with a standard 12.5x eye piece magnification range from 7.5 pixels to 32.3 pixels, enables all details to be observed without vignetting. The optional 10 pixel eye piece was used for low magnification observation. With digital photography, a photo frame eye piece can be attached to the unit to show the available shooting area, while the image can be displayed on the computer monitor. After 5 months of therapy with the rMnSOD gel formulation, the visualacuity of right eye remained unchanged (Figure 1(c)) at 9/10, while the visual acuity of left eye (Figure 1(d)) got worse at 6/10. The patient did not show any toxicity during all the therapy. rMnSOD is a recombinant protein easily administrable* in vitro* and* in vivo* and it is very active against the free radicals. The protein is very stable in solution and is able to enter cells. On the contrary, the wild type MnSOD is not administrable* in vitro* or* in vivo*. The rMnSOD enters cells through the leader peptide, which is able to recognize the estrogen receptor on the cells [13, 14]. Many researches [11–15] have demonstrated the protective action against the oxidative damage for organs and tissues of animals. The protective effects of rMnSOD gel formulation have been reported in a recent study on rabbit eyes by us [11]. In this case report, we want to highlight that the rMnSOD protects from degenerative process of PSC, compared to the current therapy with bendaline, internationally allowed [15]. Our findings suggest that rMnSOD gel formulation might be used also to protect eyes in diseases, as PSC, from oxidative damages. The patient will continue to be followed up in our clinic.

## 4. Conclusion

According to the current knowledge about the therapeutic target role of the redox balance, this case report suggests an important action of the rMnSOD which, vehicled as an ophthalmic gel, would achieve a good therapeutic efficiency without side effects. As previously demonstrated in our ophthalmologic study on rabbit eye [11], the rMnSOD is able to reduce the oxidative stress, thus preventing the worsening of PSC disease. Based on the evidence of the protective effects of rMnSOD versus PSC disease, probably due to the capacity of rMnSOD of countering free radical species, our findings suggest that rMnSOD gel formulation could be considered as an associated additional treatment for PSC. Further study will be performed before taking action.

## Abbreviations

IOP: Intraocular pressure
LECs: Lens epithelial cells
LOCSII or LOCSIII: Lens Opacification Classification System
PSCs: Posterior subcapsular cataracts
ROS: Reacting Oxygen Species
rMnSOD: Recombinant manganese superoxide dismutase
SOD: Superoxide dismutase (SOD).

## References

[1] Thylefors B. (1998). A global initiative for the elimination of avoidable blindness. *The American Journal of Ophthalmology*.

[2] Klein B. E. K., Klein R., Linton K. L. P. (1992). Prevalence of age-related lens opacities in a population: the Beaver Dam Eye Study. *Ophthalmology*.

[3] Shiels A., Hejtmancik J. F. (2007). Genetic origins of cataract. *Archives of Ophthalmology*.

[4] Zuercher J., Neidhardt J., Magyar I. (2010). Alterations of the 5′untranslated region of *SLC16A12* lead to age-related cataract. *Investigative Ophthalmology & Visual Science*.

[5] James E. R. (2007). The etiology of steroid cataract. *Journal of Ocular Pharmacology and Therapeutics*.

[6] Ates O., Alp H. H., Kocer I., Baykal O., Salman I. A. (2010). Oxidative DNA damage in patients with cataract. *Acta Ophthalmologica*.

[7] Çelik S. K., Aras N., Yildirim Ö. (2015). Glutathione S-transferase GSTM 1, null genotype may be associated with susceptibility to age-related cataract. *Advances in Clinical and Experimental Medicine*.

[8] Babizhayev M. A., Yegorov Y. E. (2014). Biomarkers of oxidative stress and cataract. novel drug delivery therapeutic strategies targeting telomere reduction and the expression of telomerase activity in the lens epithelial cells with N-acetylcarnosine lubricant eye drops: anti-cataract which helps to prevent and treat cataracts in the eyes of dogs and other animals. *Current Drug Delivery*.

[9] Khoshaman K., Yousefi R., Mohammad Tamaddon A., Saso L., Moosavi-Movahedi A. A. (2015). The impact of Hydrogen peroxide on structure, stability and functional properties of Human R12C mutant *α*a-crystallin: the imperative insights into pathomechanism of the associated congenital cataract incidence. *Free Radical Biology and Medicine*.

[10] Costagliola C., Iuliano G., Menzione M., Apponi-Battini G., Auricchio G. (1987). Effect of topical glucocorticoid administration on the protein and nonprotein sulfhydryl groups of the rabbit lens. *Ophthalmic Research*.

[11] Grumetto L., Del Prete A., Ortosecco G. (2015). Study on the protective effect of a new manganese superoxide dismutase on the microvilli of rabbit eyes exposed to UV radiation. *BioMed Research International*.

[12] Del Prete A., Loffredo C., Carderopoli A., Caparello O., Verde R., Sebastiani A. (1994). Local specific immunotherapy in allergic conjunctivitis. *Acta Ophthalmologica*.

[13] Mancini A., Borrelli A., Schiattarella A. (2006). Tumor suppressive activity of a variant isoform of manganese superoxide dismutase released by a human liposarcoma cell line. *International Journal of Cancer*.

[14] Borrelli A., Schiattarella A., Mancini R. (2016). A new hexapeptide from the leader peptide of rMnSOD enters cells through the oestrogen receptor to deliver therapeutic molecules. *Scientific Reports*.

[15] Balfour J. A., Clissold S. P. (1990). Bendazac lysine. A review of its pharmacological properties and therapeutic potential in the management of cataracts. *Drugs*.

